# Linking executive functions to distracted driving, does it differ between young and mature drivers?

**DOI:** 10.1371/journal.pone.0239596

**Published:** 2020-09-24

**Authors:** Zhi Zhang, Yingshi Guo, Rui Fu, Wei Yuan, Chang Wang

**Affiliations:** School of Automobile, Chang'an University, Xi'an, China; Tongii University, CHINA

## Abstract

Distracted driving is a leading cause of traffic accidents. Certain executive functions significantly affect the willingness of distracted driving; however, little research has compared the effects of executive functions on distracted driving behaviors in different aged populations. This study explores and compares the behavioral and cognitive processes underlying distracted driving behaviors in young and mature drivers. A total of 138 participants aged 18–65 years old completed a self-report questionnaire for measuring executive function index and distracted driving behaviors. Independent sample t-tests were conducted for executive functions (motivational drive, organization, strategic planning, impulse control, and empathy) and driving variables to examine any differences between young and mature groups. Partial correlation coefficients and z-score of these comparisons were calculated to compare the differences between age groups. Furthermore, multiple hierarchical regression models were constructed to determine the relative contributions of age, gender, and executive functions on distracted driving behaviors. Results demonstrated the following: (1) Mature drivers performed better for impulse control, the executive function index as well as the measure of distracted driving behavior than young drivers; (2) the relationships between executive functions and distracted driving behaviors did not significantly differ between young and mature drivers; (3) for both young and mature drivers, motivational drive and impulse control were found to significantly improve the prediction of distracted driving behavior in regression models. The findings emphasize that similar behavioral and cognitive processes are involved in distracted driving behavior of young and mature drivers, and can promote a single strategy for driver education and accident prevention interventions for both age groups.

## 1. Introduction

The WHO reported that about 1.35 million people of all ages were killed in road traffic accidents globally in 2016 [1]. With the rapid development of in-vehicle systems and portable electronic devices, distracted driving behaviors are among the leading causes of traffic violations. They increasingly contribute to the risk of crashes, which pose a danger to vehicle occupants, pedestrians, and bicyclists. In 2017, 2935 fatal crashes occurred on roadways in the USA that involved distraction, with 3166 people killed in motor vehicle crashes involving distracted drivers, including 599 non-occupants (pedestrians, bicyclists, and others). Additionally, 83% of drivers aged 15–59 years old that were involved in fatal crashes were distracted at the time of the crashes, and Individual factors were found to affect injury severity in traffic accidents, such as driver age, gender, etc. [2–4]. Driver distraction is a specific type of driver inattention, which may impair driving performance in terms of speed control, lane-keeping, and responding to dangerous situations [5, 6]. Various activities are identified as distracted behaviors, such as cell phone use and texting, eating, talking to other passengers, or adjusting the vehicular equipment while driving [2].

Executive functions are defined as goal-directed behaviors and are considered as a crucial attribute explaining behavioral differences among drivers [7]. Multiple aspects of executive functions, including inhibitory control, cognitive flexibility, and working memory, have been identified as neurocognitive skills related to the prefrontal cortex, and are typically assessed via behavioral indexes [8]. They play distinctive roles in the selection and allocation of attention resources and consist of a list of cognitive processes by which an individual exerts conscious control over their thoughts and actions [9]. Several researchers have attempted to link specific executive functions to self-regulation, and have suggested that temporary reductions in executive functions underlay successful self-regulation and its failure in tempting environments [10]. Many of the situational factors identified in previous studies were found to temporarily impair both self-regulation and executive functions, including alcohol intoxication [11], environmental stressors [12], and cognitive load [13]. Correspondingly, when linking these situational factors to driving, many scholars in driving research have attempted to explain risky driving behavior from the perspective of poor executive functions. Especially for young drivers, due to poor executive functions, young drivers were found to engage in high speed and large lane deviation on a driving simulator [14–17]. Additionally, findings from the study of Ross et al. demonstrated that two aspects of executive function, response inhibition and verbal working memory, were negatively associated with the standard deviation of the lateral lane position [18]. Response inhibition was also negatively related to the response to road hazards in young novice drivers. Mäntylä et al. concluded that only working memory capacity could significantly predict individual variability in lateral deviation in the lane change task for teenage novice drivers [8]. Zhang et al. found that cognitive factors (working memory capacity and response inhibition capacity) and situational factors (time pressure) had almost equal effects on responses to critical events in a simulated driving study [19].

There are also several studies conducted in other aged populations. For example, Tabibi et al. investigated three components of executive function: working memory, sustained attention, and behavioral inhibition, to explain aberrant driving behavior, driving errors, driving violations, and crashes, with the age of participants ranging from 19–49 years [20]. The results revealed inhibitory control related to aberrant driving behavior and crashes. Another study by Starkey and Isler investigated the role of executive function in explaining driving behavior in adolescent (16–18 years) and adult (25 years and over) male drivers, finding that greater working memory was associated with higher levels of self-reported risky driving and more accepting attitudes to risky driving [21]. Ledger et al. explored the relationship between cognitive functions and driving performance in younger and older drivers, obtaining a similar model for cognitive function and driving performance in both population groups [22].

As a special driving behavior, distracted driving has also been the focus of studies regarding individual executive function differences. For example, Hayashi et al. investigated the relationship between executive functions and texting while driving in college students, Texting while driving was found to be strongly correlated with a low level of impulse control [23]. A study by Louie and Mouloua found that higher working memory capacity could improve the distracted driving performance of undergraduate students [24]. These studies mainly focused on younger population, as they still developed for executive functions until the age of 25 [9]. In addition, Pope et al. investigated the relationship between age, executive function, and self-reported distracted driving behaviors. The results revealed that global executive difficulty was significantly related to distracted driving behaviors, and the relationship between age and distracted driving behavior was partially mediated by global executive difficulty [25]. Previous studies have suggested risky behaviors, such as the willingness to engage in distracted driving, are dependent on the balance between cognitive control and socioemotional systems. The cognitive control system consists of multiple executive functions, such as planning, working memory, and impulse control, which enables the prevention of risky behavior and adaptation to task goals. Socioemotional systems are involved in response to emotions, thrills, social cues, and environmental sensations, which can lead to risk-taking [26, 27]. This suggests that mature drivers (25 years old and over) may also need to employ the cognitive control system to withstand willingness to engage in distracted driving as young drivers (below 25 years old) do. However, it is not clear whether the role of specific executive functions differ considerably in different age groups.

Therefore, further research is required to analyze the effect of executive function on distracted driving behaviors in young and mature drivers. In the present study, the correlations between executive functions and distracted driving behaviors are re-examined in different aged populations. Executive Function Index (EFI) has been developed to assess the level of executive functions in a normal population and verified as highly effective in the study of driving behaviors [28, 29], and is used in this study. A distracted driving questionnaire, including various distracted behaviors in daily life, is applied for the assessment of distracted driving behaviors [30]. Furthermore, according to the results of previous research, two hypotheses are proposed. The first is that mature drivers perform better in executive functions and distracted driving behavior than young drivers. The second is that the effect of executive functions on distracted driving behavior is comparable for young and mature drivers.

## 2. Material and methods

### 2.1. Participants

A total of 151 participants with a valid driving license and at least two years of driving experience were recruited by sampling from a volunteer database of the key laboratory of the Ministry of Transport of China at Chang’an University. All subjects had driven almost every day in the last 12 months. Thirteen participants were excluded due to incomplete questionnaires, and the data of the remaining 138 participants (70 male and 68 female, 18–65 years old) were analyzed. The ethical protocol of the present study was approved by the Institutional Review Board at Chang’an University.

### 2.2. Procedure

Sessions were conducted in the lab. After providing written informed consent, all recruited participants were explicitly informed to complete a questionnaire that involved demographic information, executive function, and driving behaviors. The questionnaires took approximately 30 min to complete, and all participants were monetarily reimbursed for their completion of the study.

#### 2.2.1. Demographic and driving-related questionnaires

Participants completed a basic demographic questionnaire that included questions about age, gender, and driving years. They then completed a driving questionnaire, which included nine questions related to the participant’s distracted behaviors while driving, such as drinking, eating, cell phone use, being lost in thought, and texting [30]. Participants were instructed to report the frequency in which they engaged in each behavior over the course of one week, and the questions were presented using the following format as an example “how many times did you use a hand-free phone to talk while driving in the last week?”. A composite score was obtained by summing the number of all distracted behaviors [25]. The internal consistency of driving questionnaire was determined as acceptable (Cronbach’s alpha = 0.82).

#### 2.2.2. Executive function index

Individual executive function was assessed according to EFI [28], which is a self-reported measure with 27 questions on real-world behavioral disruptions that uses a five-point Likert scale ranging from 1 (never) to 5 (always). The EFI questionnaire is comprised of five subcategories, namely motivational drive (MD), organization (ORG), strategic planning (SP), impulse control (IC), and empathy (EM). Different questions could represent one of the five aspects of executive function, such as the use of “Interested in new things” to reflect the domain of motivational drive, “Save money regularly” to reflect the domain of organization, “Anticipate consequences of actions” to reflect the domain of strategic planning, “Lose my temper when upset” to reflect the domain of impulse control, and “Concern for others” to reflect the domain of empathy. Several negatively-worded items were inverted, and the five composite scores were summed together to obtain the total EFI score, indicating the level of executive function [23]. The internal consistency of the total EFI score was acceptable in this study (Cronbach’s alpha = 0.80) according to the conclusion of the previous studies [31, 32].

### 2.3. Data analysis

In the present study, a composite score of distracted driving behavior was investigated as a dependent variable. Similar to previous studies, to examine the differences between two age groups, all participants were classified into a young group (younger than 25 years) or mature group (25 years and above) [33]. About 50.7% of the participants were classified as the young group and the remaining 49.3% as the mature group. No significant age differences were found between male and female participants (F(1, 136) = 1.337, p = 0.250). First, independent sample t-tests were conducted for five executive functions and distracted driving variables to examine any differences between the young and mature groups. Second, to compare and determine the differences between younger and older drivers, correlation tests between each executive function score and distracted driving behavior were performed. The correlation coefficients were calculated separately for young drivers and mature drivers, then a z-score of the comparisons between age groups was calculated to identify the statistical difference of the correlation coefficients [22]. Finally, to further determine the relative contributions of age, gender, and the five executive functions on distracted driving behaviors, multiple hierarchical regression modeling was carried out using a forward step-wise strategy. The computer software SPSS 22.0 was used in the present study.

## 3. Results

### 3.1. Comparison of young and mature drivers for executive functions and distracted driving

**Fig 1:**
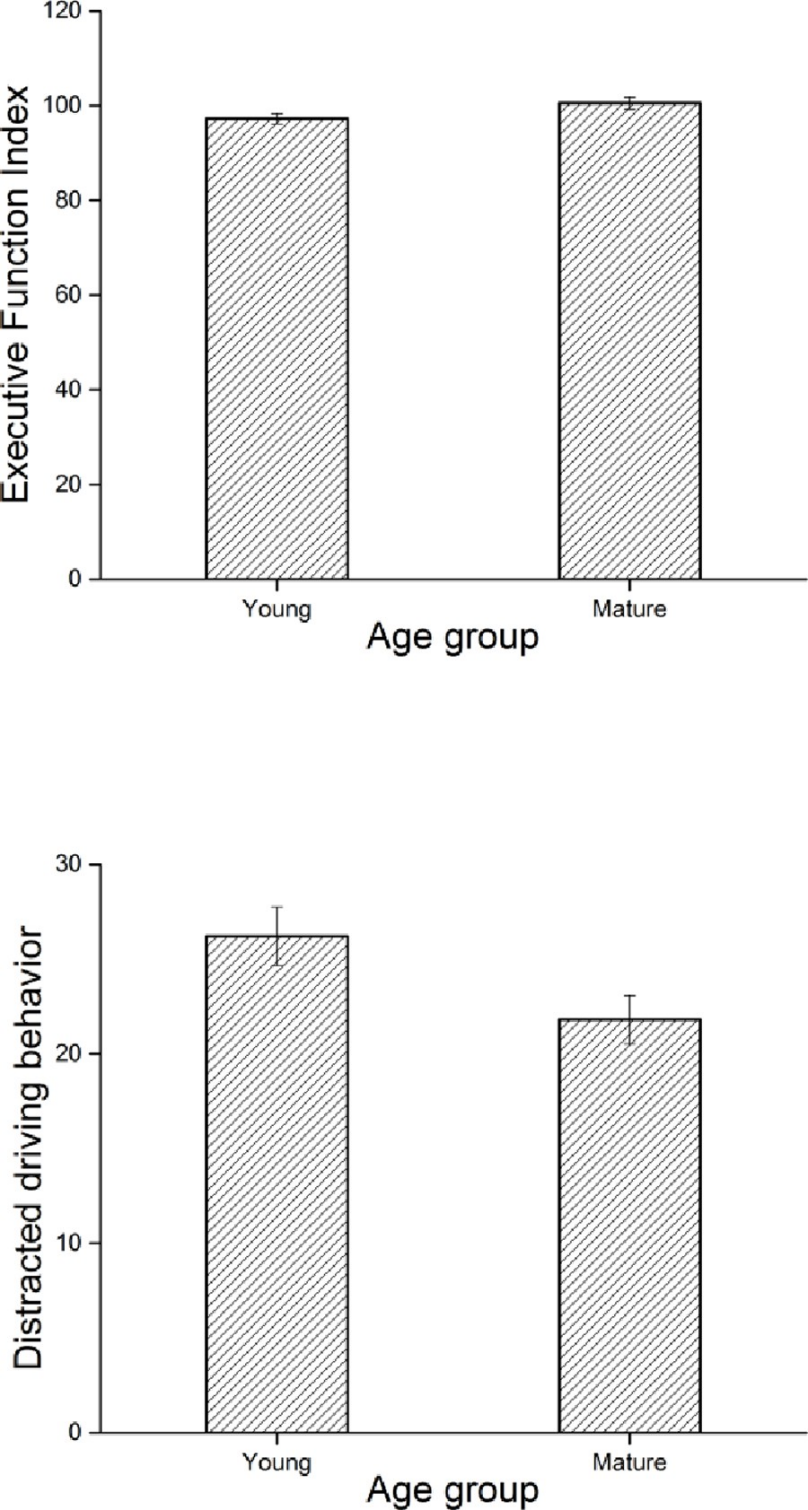
The score of EFI total and distracted driving behavior by age groups (error bars indicate the standard error).

Results of independent sample t-tests for major variables are presented in Table 1, revealing that mature drivers had significantly higher IC (p = 0.004) and EFI (p = 0.049) scores than younger drivers. A significant age difference was also found in driving performance, with mature drivers performing better on the measure of distracted driving behavior (p = 0.030) than young drivers. The score of EFI total and distracted driving behavior for young and mature groups is provided in Fig 1.

**Table 1:** Independent samples t-tests by the variable of age.

Variables	Young		Mature		T	Sig.
	Mean	Std.	Mean	Std.		
1. EM	22.60	3.976	23.53	3.462	-1.463	0.146
2. ORG	17.86	3.657	17.97	3.434	-0.188	0.851
3. SP	20.81	2.024	20.59	2.261	0.619	0.537
4. IC	21.56	3.412	23.16	2.920	-2.964	**0.004**
5. MD	14.41	3.903	15.29	4.832	-1.178	0.241
6. EFI	97.24	9.204	100.54	10.306	-1.986	**0.049**
7. Distracted driving	26.21	12.993	21.81	10.517	2.186	**0.030**

NOTE. Significant effects are shown in bold.

### 3.2. Association of executive functions with distracted driving for young and mature drivers

Partial correlation analysis was conducted between each executive function variable and distracted driving behavior while controlling the variables of age and gender (see Table 2). For young drivers, distracted driving behavior was significantly negatively correlated with scores of MD (r = -0.469, p < 0.001) and EFI (r = -0.305, p < 0.05), and a marginally significant correlation was found between distracted driving behavior and the IC score. There was no significant relation between distracted driving behavior and other executive function variables, such as EM, ORG, and SP. For mature drivers, distracted driving behavior was significantly negatively correlated with scores of IC (r = -0.274, p < 0.05), MD (r = -0.395, p < 0.01), and EFI (r = -0.262, p < 0.05), while no significant correlation was found between distracted driving behavior and EM, ORG, or SP.

**Table 2:** Partial correlations among major variables.

Variables	1	2	3	4	5	6	7
1. Distracted driving							
Young drivers		-0.305*	-0.135	0.140	-0.037	-0.235	-0.469***
Mature drivers		-0.262*	-0.042	0.094	-0.078	-0.274*	-0.395**
2. EFI							
Young drivers			0.711***	0.620***	0.527***	0.520***	0.273*
Mature drivers			0.688***	0.607***	0.659***	0.568***	0.553***
3. EM							
Young drivers				0.497***	0.098	0.239	-0.108
Mature drivers				0.481***	0.216	0.200	0.182
4. ORG							
Young drivers					0.336**	-0.015	-0.176
Mature drivers					0.543***	0.180	-0.125
5. SP							
Young drivers						0.377**	-0.044
Mature drivers						0.425***	0.134
6. IC							
Young drivers							-0.093
Mature drivers							0.129
7. MD							

NOTE.

* p < 0.05.

** p < 0.01.

*** p < 0.001

### 3.3. Comparison of the correlations

To compare the relationships between each executive function variable and distracted driving behavior, the z-score of the correlation coefficient comparisons between young and mature groups was calculated, as shown in Table 3. The results revealed the z-scores of these comparisons were less than 1.96 and greater than -1.96 (p > 0.05), suggesting that the relationship between distracted driving behavior and executive functions did not significantly differ between young and mature drivers.

**Table 3:** Comparison of independent correlation coefficient (z-score).

Variables	EFI	EM	ORG	SP	IC	MD
Distracted driving	1.226	-0.377	-0.401	0.412	1.377	1.891

### 3.4. Hierarchical regression modeling

To further determine the relative contributions of different predictors on distracted driving behavior, a multiple hierarchical regression model (full model) and a forward step-wise regression model (final model) were performed, full model is with all predictors, and final model is only with the predictors of a significance level less than 0.05. Tables 4 and 5 summarize the regression models for predicting distracted driving behavior.

**Table 4:** Multiple regression models among young drivers.

	Full model			Final Model			
Predictor	Beta	R^2^	Sig.	Beta	R^2^	ΔR^2^	Sig.
		0.336	**0.000**		0.291		**0.000**
MD	-0.344		**0.000**	-0.471		0.202	**0.000**
IC	-0.245		**0.045**	-0.299		0.089	**0.005**
EM	-0.231		0.073				
ORG	0.186		0.169				
SP	0.019		0.879				

NOTE. R^2^ = explained variance, ΔR^2^ = increased explained variance, Sig. = Significance level.

Significant effects are shown in bold.

**Table 5:** Multiple regression models among mature drivers.

	Full model			Final Model			
Predictor	Beta	R^2^	Sig.	Beta	R^2^	ΔR^2^	Sig.
		0.260	0.002		0.248		**0.000**
MD	-0.391		**0.002**	-0.402		0.193	**0.000**
IC	-0.272		**0.031**	-0.239		0.056	**0.032**
ORG	0.085		0.588				
SP	0.036		0.808				
EM	0.013		0.924				

NOTE. R^2^ = explained variance, ΔR^2^ = increased explained variance, Sig. = Significance level.

Significant effects are shown in bold.

As seen in Table 4, the full hierarchical regression model among young drivers was found to be significant, F(5, 64) = 6.466, p < 0.001, and accounted for 33.6% of the total variance. The full model revealed that IC (p = 0.045) and MD (p < 0.001) significantly contributed to the predictive model, and the negative coefficients of IC (-0.245) and MD (-0.344) suggested that the frequency of distracted driving behavior increased as the score of IC or MD decreased. Other predictors, including EM, ORG, and SP, were not found to be significant in the full model. The final model revealed the relative contribution of significant predictors on the distracted driving behavior of young drivers. MD significantly contributed to the model at step one, F(1, 68) = 17.205, p < 0.001, accounting for 20.2% of the total variance. The introduction of the IC variable in step two significantly improved the model, F-change(2, 67) = 8.422, p = 0.005, accounting for 8.1% of the total variance. Together, this model was found to be significant, F(2, 67) = 13.752, p < 0.001, and accounts for 29.1% of the total variance.

As seen in Table 5, the full hierarchical regression model among mature drivers was also found to be significant, F(5, 62) = 4.368, p = 0.002, accounting for 26.0% of the total variance. The full model revealed that IC (p = 0.031) and MD (p = 0.002) significantly contributed to the predictive model, the negative coefficients of IC (-0.272) and MD (-0.391) suggested that the frequency of distracted driving behavior increased as the score of IC or MD decreased. Other predictors, including EM, ORG, and SP, were not found to be significant in the full model. The final model revealed the relative contribution of significant predictors on the distracted driving behavior of mature drivers, MD did significantly contributed to the model at step one, F(1, 66) = 15.745, p < 0.001, accounting for 19.3% of the total variance. The introduction of the IC variable in step two significantly improved the model, F-change (2, 65) = 4.819, p = 0.032, accounting for 5.6% of the total variance. Together, this model was found to be significant, F(2, 65) = 10.738, p < 0.001, and accounts for 24.8% of the total variance.

## 4. Discussion

The current study aimed to explore and compare the behavioral and cognitive processes underlying distracted driving behaviors in young and mature drivers. Five subcategories of executive function, including motivational drive, organization, strategic planning, impulse control, and empathy, were investigated as independent variables, and distracted driving behaviors were investigated as dependent variables. The first hypothesis regarding mature drivers performing better in executive functions and distracted driving behavior than young drivers was partly supported. In particular, mature drivers had significantly higher IC and EFI scores than young drivers, and a notable age difference was also found in the measure of distracted driving behavior. The results of our study also supported the second hypothesis that the effect of executive functions on distracted driving behavior would be comparable for both age groups, with an insignificant z-score of the correlation coefficient comparisons between young and mature groups. Furthermore, the relative influence of specific executive function on distracted driving behavior was found to be significantly different, IC and MD contributed more to distracted driving behavior than others in the hierarchical regression models.

This study examined the differences between young and mature groups in executive functions and distracted driving behaviors. Evidence was found that there were significant differences in impulse control and distracted driving behaviors. In the majority of cases, mature drivers displayed better performances in executive functions and distracted driving behaviors than young drivers. This provides evidence to suggest that, as executive functions are still developing for young people, they fail to adapt their behaviors to a task goal in tempting environments [10, 34]. For example, texting while driving is particularly pervasive among young drivers. Many drivers still engage in texting while driving, even though they are aware of its negative consequences [35]. Based on the results of this study, poor impulse control is an important influence in this decision-making process.

To compare young and mature drivers, partial correlation analysis between each of executive function variables and distracted driving behavior was conducted, with a significant relationship between EFI and distracted driving behavior found in both young and mature drivers. Specifically, the relationship suggested that for both age groups, as motivational drive worsened, so too did the measure of distracted driving behavior. The relationship between impulse control and distracted driving behavior for mature drivers was found to be significant, but it did not exist for young drivers. These results were consistent with previous studies that found a significant correlation between executive function difficulty and distracted driving in all ages [25]. Furthermore, the z-scores of the correlation coefficient comparison between the two groups were calculated, with all pairs being found to differ insignificantly. This finding supports the conclusion that the effect of executive functions on distracted driving behavior is comparable for both age groups, and that similar behavioral and cognitive processes are involved in distracted driving behavior of young and mature drivers. This also suggests that a similar intervention strategy may be useful for both young and mature drivers to reduce distracted driving behaviors.

The conclusions of previous studies were also replicated in this research by illustrating the significant effect of executive function measured by the EFI score on distracted driving behaviors [23, 36]. Moreover, separate assessments of different executive functions can lead to further understanding of the differences between young and mature drivers. Previous studies found that distracted driving behavior (texting while driving) was only significantly correlated with impulse control in young drivers [36]. The findings of our study revealed that both impulse control and motivational drive contributed to distracted driving behaviors. This inconsistent conclusion can be attributed to differences in the measurement of distracted driving behaviors. In this study, we adopted more distracted behaviors in daily life, rather than one distracted behavior as an indicator. This result is interesting because it suggests that distracted driving is not only a form of impulsive behavior due to a lack of inhibitory control but also a joint function of inhibitory control and motivational drive. Motivational drive is identified as behavioral drive or interest in novelty, individuals with lower levels of motivational drive show more apathy or no interest in the primary driving task [28]. Furthermore, as orbitofrontal circuits mediate self-inhibition, whereas medial prefrontal circuits mediate motivational aspects of behavior [37], the findings possibly indicate that a driver’s willingness to engage in distracted behaviors is regulated by multiple parts of the brain, and individuals with less inhibitory control or motivational control are more likely to engage in distracted driving behaviors.

Several limitations of the present study should be noted. First, the sample size was relatively small, and a larger sample of drivers is needed to further test the external validity of the present findings. Second, due to the inherent drawback of self-reported measures, the accuracy of measures of executive functions and distracted driving depends on the drivers’ self-evaluation of their own behavior over time. Performance-based methods, such as operation span and go/no-go tasks, should be employed to assess executive functions in subsequent research. Furthermore, using observational data of distracted behaviors in real-world driving to assess distracted driving also warrants further investigation. Third, as the current research only focuses on sample participants aged 18–65 years old, the generalizability of the findings is limited to an older group over 65 years old. It would be interesting to determine whether the relationships between executive functions and distracted driving apply to older drivers in future studies.

## 5. Conclusion

Executive functions may be important predictors of many real-world behaviors. This study explored and compared the roles of executive functions in distracted driving in young and mature drivers. The results revealed that specific executive function has differentiated links with distracted driving behaviors as motivational drive and impulse control contribute more to distracted driving behavior than other executive functions for both young and mature drivers. These findings provide a unique opportunity to target drivers who are more likely to engage in distracted driving behaviors. The results also revealed that the relationships between distracted driving behavior and executive functions did not significantly differ between young and mature drivers. This indicates that a similar intervention strategy may be useful for both young and mature drivers to reduce distracted driving behaviors.

In summary, these findings may have two aspects of implication. Theoretically, using executive functions as individual characteristics, our study confirmed the generalizability and similarity of the effect of executive functions on distracted driving behaviors in both young and mature adults. Practically, our findings can assist in the development of driver education and accident prevention intervention. For instance, knowledge of the correlation between executive functions and distracted driving behaviors can be helpful in the screening of professional drivers. It can also be used in the education and training of novice drivers. Further, the current results can contribute to the development of ADAS, such as a vehicular warning system for driver distractions. A vehicular warning system could warn drivers in a positive and friendly way by considering the relationship between executive functions and distracted driving for moderating negative aspects of poor executive functions in distracted driving.

## Supporting information

S1 Data (XLS)

S1 Questionnaires (DOCX)

## References

[1] World Health Organization, 2018. Global Status Report on Road Safety 2018: Summary (No. WHO/NMH/NVI/18.20). World Health Organization.

[2] National Highway Traffic Safety Administration [NHTSA]. Distracted Driving in Fatal Crashes 2017. 2019. Retrieved from <https://crashstats.nhtsa.dot.gov/Api/Public/ViewPublication/812700>.

[3] Chen, Song, Ma. Investigation on the Injury Severity of Drivers in Rear-End Collisions Between Cars Using a Random Parameters Bivariate Ordered Probit Model. International Journal of Environmental Research and Public Health. 2019; 16(14):2632 10.3390/ijerph16142632 31340600 PMC6678079

[4] Zeng Qiang, Hao Wei, Lee Jaeyoung, Chen Feng. Investigating the Impacts of Real-Time Weather Conditions on Freeway Crash Severity: A Bayesian Spatial Analysis. International Journal of Environmental Research and Public Health, 2020, 17(8): 2768 10.3390/ijerph17082768 32316427 PMC7215785

[5] Zhang Z., Guo Y., Yuan W., & Wang C. The impact of cognitive distraction on driver perception response time under different levels of situational urgency. IEEE Access. 2019; 7: 184572–184580. 10.1109/ACCESS.2019.2960830

[6] Wang C., Li Z., Fu R., Guo Y., Yuan W. What is the difference in driver’s lateral control ability during naturalistic distracted driving and normal driving? A case study on a real highway. Accident Analysis & Prevention. 2019; 125: 98–105. 10.1016/j.aap.2019.01.030 30738295

[7] Aksan N., Anderson S. W., Dawson J., Uc E., Rizzo M. Cognitive functioning differentially predicts different dimensions of older drivers' on-road safety. Accident Analysis & Prevention. 2015; 75: 236–244. 10.1016/j.aap.2014.12.007 25525974 PMC4386614

[8] Mäntylä T., Karlsson M. J., Marklund M. Executive control functions in simulated driving. Applied neuropsychology. 2009; 16(1): 11–18. 10.1080/09084280802644086 19205943

[9] Romer D., Betancourt L. M., Brodsky N. L., Giannetta J. M., Yang W., Hurt H. Does adolescent risk taking imply weak executive function? A prospective study of relations between working memory performance, impulsivity, and risk taking in early adolescence. Developmental science. 2011; 14(5): 1119–1133. 10.1111/j.1467-7687.2011.01061.x 21884327 PMC3177153

[10] Hofmann W., Schmeichel B.J., Baddeley A.D. Executive functions and self-regulation. Trends in Cognitive Sciences. 2012; 16(3): 174–180. 10.1016/j.tics.2012.01.006 22336729

[11] Hofmann W., Friese M. Impulses got the better of me: Alcohol moderates the influence of implicit attitudes toward food cues on eating behavior. J Abnorm Psychol. 2008; 117: 420–427. 10.1037/0021-843X.117.2.420 18489218

[12] Beilock S.L., Carr T.H. When high-powered people fail—Working memory and "choking under pressure'' in math. Psychological Science. 2005; 16(2): 101–105. 10.1111/j.0956-7976.2005.00789.x 15686575

[13] Friese M., Hofmann W., Wnke M. When impulses take over: moderated predictive validity of explicit and implicit attitude measures in predicting food choice and consumption behavior. British Journal of Social Psychology. 2007; 47: 397–419. 10.1348/014466607X241540 17880753

[14] Zicat E., Bennett J. M., Chekaluk E., Batchelor J. Cognitive function and young drivers: the relationship between driving, attitudes, personality and cognition. Transportation research part F: traffic psychology and behaviour. 2018; 55: 341–352. 10.1016/j.trf.2018.03.013

[15] Albert D.A., Ouimet M.C., Jarret J., Cloutier M.S., Paquette M., Badeau N., et al Linking mind wandering tendency to risky driving in young male drivers. Accident Analysis & Prevention. 2018; 111: 125–132. 10.1016/j.aap.2017.11.019 29197692

[16] Brown T.G., Ouimet M.C., Eldeb M., Tremblay J., Vingilis E., Nadeau L., et al Personality, executive control, and neurobiological characteristics associated with different forms of risky driving. PloS one. 2016; 11(2): 150–227. 10.1371/journal.pone.0150227 26910345 PMC4766103

[17] Žardeckaitė-Matulaitienė K., Pranckevičienė A., Šeibokaitė L., Endriulaitienė A. Cognitive functions and simulated risky driving in a sample of the young drivers. In Transport means-2013: proceedings of the 17th international conference, 2013; 29–32.

[18] Ross V., Jongen E., Brijs T., Ruiter R., Brijs K., Wets G. The relation between cognitive control and risky driving in young novice drivers. Applied Neuropsychology: Adult. 2015; 22(1): 61–72. 10.1080/23279095.2013.838958 25529593

[19] Zhang Z., Guo Y., Fu R., Yuan W. Yang G. Do situational or cognitive factors contribute more to risky driving? A simulated driving study. Cognition, Technology & Work. 2020 10.1007/s10111-020-00630-3

[20] Tabibi Z., Borzabadi H.H., Stavrinos D., Mashhadi A. Predicting aberrant driving behaviour: the role of executive function. Transportation Research Part F: Traffic Psychology and Behaviour. 2015; 34: 18–28. 10.1016/j.trf.2015.07.015

[21] Starkey N.J., Isler R.B. The role of executive function, personality and attitudes to risks in explaining self-reported driving behaviour in adolescent and adult male drivers. Transportation research part F: traffic psychology and behaviour. 2016; 38: 127–136. 10.1016/j.trf.2016.01.013

[22] Ledger S., Bennett J. M., Chekaluk E., Batchelor J. Cognitive function and driving: important for young and old alike. Transportation research part F: traffic psychology and behaviour. 2019; 60: 262–273. 10.1016/j.trf.2018.10.024

[23] Hayashi Y., Rivera E. A., Modic J.G., Forema A M., Wirth O. Texting while driving, executive function, and impulsivity in college students. Accident Analysis & Prevention. 2017; 102: 72–80. 10.1016/j.aap.2017.02.016 28267655 PMC6481653

[24] Louie J.F., Mouloua M. Predicting distracted driving: The role of individual differences in working memory. Applied Ergonomics. 2019; 74: 154–161. 10.1016/j.apergo.2018.07.004 30487094

[25] Pope C.N., Bell T.R., Stavrinos D. Mechanisms behind distracted driving behavior: The role of age and executive function in the engagement of distracted driving. Accident Analysis & Prevention. 2017; 98: 123–129. 10.1016/j.aap.2016.09.030 27716494 PMC5167635

[26] Steinberg L. A social neuroscience perspective on adolescent risk-taking. Developmental Review. 2008; 28(1): 78–106. 10.1016/j.dr.2007.08.002 18509515 PMC2396566

[27] Groeger. Understanding driving: Applying cognitive psychology to a complex everyday task, Routledge. 2013.

[28] Spinella M. Self-rated executive function: development of the executive function index. International Journal of Neuroscience. 2005; 115(5): 649–667. 10.1080/00207450590524304 15823930

[29] Roth R.M., Lance C. E., Isquith P. K., Fischer A.S., Giancola P.R. Confirmatory factor analysis of the behavior rating inventory of executive function-adult version in healthy adults and application to attention-deficit/hyperactivity disorder. Archives of clinical neuropsycholog. 2013; 28(5): 425–434. 10.1093/arclin/act031 23676185 PMC3711374

[30] Welburn S. C., Garner A. A., Schwartz M., Stavrinos D. Developing a self-report measure of distracted driving in young adults. In Poster presented at the 2010 University of Alabama at Birmingham Expo for Undergraduate Research. 2010.

[31] Rucker D. D., Preacher K. J., Tormala Z. L., Petty R. E. Mediation analysis in social psychology: Current practices and new recommendations. Social and Personality Psychology Compass. 2011; 5(6): 359–371. 10.1111/j.1751-9004.2011.00355.x

[32] Meyers L. S., Gams G., Guarino A.J. Applied multivariate research: Design and interpretation. Sage publications 2016.

[33] Yadav A.K., Velaga N.R. Modelling the relationship between different Blood Alcohol Concentrations and reaction time of young and mature drivers. Transportation Research Part F: Traffic Psychology and Behaviour. 2019; 64: 227–245. 10.1016/j.trf.2019.05.011

[34] Friedman N.P., et al Individual differences in executive functions are almost entirely genetic in origin. Journal of Experimental Psychology: General. 2008; 137(2): 201–225. 10.1037/0096-3445.137.2.201 18473654 PMC2762790

[35] Atchley P., Atwood S., Boulton A. The choice to text and drive in younger drivers: behavior may shape attitude. Accident Analysis & Prevention. 2011; 43: 134–142. 10.1016/j.aap.2010.08.003 21094307

[36] Hayashi Y., Foreman A. M., Friedel J. E., Wirth O. Executive function and dangerous driving behaviors in young drivers. Transportation research part F: traffic psychology and behaviour. 2018; 52, 51–61. 10.1016/j.trf.2017.11.007 31024220 PMC6477690

[37] Teki S., Cummings J. L. Frontal-subcortical neuronal circuits and clinical neuropsychiatry: an update. Journal of psychosomatic research. 2002; 53(2): 647–654. 10.1016/s0022-3999(02)00428-2 12169339

