# Supplementary material for: Linking executive functions to distracted driving, does it differ between young and mature drivers?
Source: PLoS One. 2020 Sep 24;15(9):e0239596. doi: 10.1371/journal.pone.0239596 (PMC7514019; doi:10.1371/journal.pone.0239596)
Supplement: S1 Questionnaires — (DOCX) [file pone.0239596.s002.docx]

Your age: Sex: Driving years: Occupation:

Driving days in the last year: Driving days in the last week:

**Part one**

Please write the corresponding number that coincide with your situation for each question. Thank you very much!

1-not at all, 2- occasionally, 3- sometimes, 4- often 5- always

1.Concern for others

2.Help others in need

3.Takes others’ feelings into account

4.Protective towards a friends

5.Dislikes actions or words hurting others

6.Socially aggressive stance

7. Organized person

8. Save money regularly

9.Self-monitor for mistakes

10.Plan for the future

11.Use of memory strategies

12.Anticipate consequences of actions

13.Learn from mistakes

14. Trouble summing information for decisions

15.Distractibility

16.Lost track of what I’m doing

17. Mix up the sequences of actions

18.Trouble doing two things at once

19.Socially embarrassing behavior

20. Inappropriate sexual behavior

21.Use obscenities

22. Maladaptive risk taking

23.Lose my temper when upset

24.Interested in new things

25. Energetic person

26.Have enthusiasm

27.Inactivity

**Part two**

How many times did you engage in the following behavior while driving in the last week? Please respond with a whole number for each behavior. Thank you very much!

1. drinking

2. eating

3. talking/singing while no other passengers are present

4. reaching without taking your eyes off the road

5. driving lost in thought

6. talking on a hands-held cell phone

7. talking on a hands-free cell phone

8. using a GPS navigation system

9. sending or receiving a text message on a cell phone
